# Are working memory and behavioral attention equally important for both reading and listening comprehension? A developmental comparison

**DOI:** 10.1007/s11145-018-9840-y

**Published:** 2018-05-07

**Authors:** Language and Reading Research Consortium, Laura M. Justice, Richard Lomax, Ann O’Connell, Jill Pentimonti, Stephen A. Petrill, Shayne B. Piasta, Shelley Gray, Maria Adelaida Restrepo, Kate Cain, Hugh Catts, Mindy Bridges, Diane Nielsen, Tiffany Hogan, Jim Bovaird, J. Ron Nelson, H. Jiang, K. Farquharson

**Affiliations:** 1 Ohio State University, Columbus, USA; 2 Emerson College, Boston, USA

**Keywords:** Reading comprehension, Listening comprehension, Working memory, Attention, Word reading

## Abstract

We investigated the extent to which working memory and behavioral attention predicted reading and listening comprehension in grades 1 through 3 and, whether their relative contributions differed by modality and grade. Separate grade samples (*N* = 370; *n*s = 125, 123, and 122 for grades 1, 2, and 3 respectively) completed multiple measures of word reading, working memory, and parallel measures of reading and listening comprehension. Teachers and parents provided behavioral attention ratings. Concurrently, working memory was more important for listening than for reading comprehension and predicted significant variance in both modalities across grades, after controlling for background measures and behavioral attention ratings. For both modalities, working memory explained the greatest proportion of variance in grade 3. Behavioral attention predicted variance in grades 1 and 2 for reading comprehension and all grades for listening comprehension. Subsidiary analyses demonstrated that the influence of working memory and behavioral attention on reading comprehension was indirect, through word reading and listening comprehension both concurrently and also longitudinally between grades 1–3. These findings indicate that delivery of classroom materials orally will not always be beneficial to the young beginner reader or one who struggles with word decoding, and that children with poor working memory/attention may require additional support to access meaning from both written and spoken text.

## Introduction

We build on research that has focused on a ‘cognitive view’ of text comprehension, examining the role of working memory and attention and how they influence reading and listening comprehension in the early grades (Cain & Bignell, 2014; Cain, Oakhill, & Bryant, 2004; Kendeou, van den Broek, Helder, & Karlsson, 2014; Sesma, Mahone, Levine, Eason, & Cutting, 2009). Problems with memory and attention are associated with poor educational attainment (Gathercole, Alloway, Willis, & Adams, 2006) and, in particular, poor literacy outcomes (Brock & Knapp, 1996; Miller et al. 2013). It is well established that language skills are critical for success in reading comprehension and educational outcomes (Cain & Oakhill, 2006; Catts, Adlof, & Weismer, 2006; Oakhill & Cain, 2012). By focusing on cognitive skills, our findings broaden our understanding of reading and listening comprehension outcomes, and speak to best practice in educational support for children with literacy difficulties and the development of effective curricula for all.

Successful comprehension of written and spoken text results in the construction of a representation of the text’s meaning, rather than a verbatim record (Zwaan & Radvansky, 1998). This representation is typically referred to as a *mental model* or a *situation model* (Johnson-Laird, 1983; Kintsch, 1998). As we read or listen to text, word meanings are retrieved, they are grouped into meaningful grammatical units, and higher-level language skills such as comprehension monitoring and inference making are engaged to construct a unified and coherent mental model (Kendeou et al. 2014; Oakhill & Cain, 2012; Perfetti, Stafura, & Adlof, 2013). These oral language skills are fundamental to successful reading comprehension: Measures of vocabulary and grammar knowledge, and the ability to generate inferences and monitor understanding of text predict concurrent and longitudinal reading comprehension performance (Kendeou, van den Broek, White, & Lynch, 2009; Oakhill & Cain, 2012; Verhoeven & van Leeuwe, 2008) and are associated with poor reading comprehension (Cain & Oakhill, 2006; Catts et al. 2006; Nation, Clarke, Marshall, & Durand, 2004).

The construction of the mental model happens in real time. As each new word or phrase is processed, the reader actively strives to integrate that information into the current mental model of that text. In doing so, the structure and content of the mental model is constantly revised and refined and provides the context for interpreting the next word, phrase, or event. Effective execution and coordination of these language skills is dependent on working memory, the workspace where the mental model is constructed (Cain, Oakhill, & Bryant, 2004). In the spirit of Baddeley and Hitch’s working memory model (Baddeley & Hitch, 1974), we focus here on verbal (rather than visuo-spatial) working memory resources that support the storage of verbal (symbolic) information while this is actively processed and manipulated for complex cognitive tasks, such as reading for meaning (Cain et al. 2004; Gathercole, Brown, & Pickering, 2003; Kendeou et al. 2014). Performance on short-term memory span measures, which assess storage, are predictive of word reading (Gathercole & Baddeley, 1993). When considering measures of working memory that tap both storage and processing, for example listening or reading span tasks, working memory scores are associated with reading comprehension outcomes in children and adults (Cain et al., 2004; Carretti, Borella, Cornoldi, & de Beni, 2009; Daneman & Merikle, 1996; Locascio, Mahone, Eason, & Cutting, 2010; Seigneuric, Ehrlich, Oakhill, & Yuill, 2000) and concurrent listening comprehension in 6-year-olds (Florit, Roch, & Levorato, 2014; Kim, 2016). Working memory also predicts reading comprehension over time (Seigneuric & Ehrlich, 2005).

Attention, and specifically the allocation of appropriate attention resources, will also influence the quality of the mental model that is constructed (Kendeou et al. 2014). The construct of attention is wide ranging: measures of cognitive attention can assess sustained attention, selective attention, and also divided attention, and attention can be assessed with both ratings scales and objective tests (Swanson, 2011). Although attention is related to working memory, the correlations for both ratings scales and objective tests with working memory are typically moderate (e.g., Swanson, 2011). In relation to attention and comprehension, several studies have demonstrated a relation between ADHD and poor reading and listening comprehension (Brock & Knapp, 1996; Martinussen & Mackenzie, 2015; McInnes, Humphries, Hogg-Johnson, & Tannock, 2003; Redmond, Ash, & Hogan, 2015). This is most likely due to children with ADHD having fewer cognitive resources to allocate to the integration of ideas within a text and the construction of the mental model, evidenced by poorer recall of the central ideas in a text (Miller et al. 2013). In work with typically developing children, attention influences 6-year-olds’ concurrent listening comprehension indirectly, through its relation with key foundational language skills (Kim, 2016). In sum, previous research suggests that both working memory and attention may influence the quality of text comprehension. In this study we sought to determine if measures of working memory and attention were predictive of both reading and listening comprehension in an unselected non-clinical population to broaden these findings.

### Reading and listening comprehension

Word reading enables reading comprehension and is the critical limiter of this ability in young children (Murphy, Language and Reading Research Consortium (LARRC), & Farquharson, 2016; Storch & Whitehurst, 2002). Poor word reading is also a source of reading comprehension difficulties (Gough and Tunmer 1986). For that reason, it has been recommended that a comparison of reading and listening comprehension will help to differentiate children with literacy difficulties that are related to poor word reading from those who have literacy difficulties despite adequate word reading (Keenan, Betjemann, Wadsworth, DeFries, & Olson, 2006). That view rests on the assumption that the primary difference between the two modalities is whether words have to be visually decoded or not. However, written and spoken text may differ also in the cognitive processing demands and support that each type of modality entails (Aaron, Joshi, Palmer, Smith, & Kirby, 2002), which might influence the contribution of working memory and attention to performance in each modality.

When comprehending a written text, the reader determines the pace of delivery and can re-read to check for meaning; for a spoken text, the speaker determines the pace and the listener cannot review the text once heard. Thus, a difficulty with accurately representing the information in working memory and/or an inability to focus or sustain attention may result in poor comprehension (Aaron et al. 2002). In addition, visually presented text may help the reader to focus attention and minimize distractibility, because each word needs to be decoded (Aaron et al. 2002). For these reasons, working memory and attention may be more strongly predictive of listening than reading comprehension.

There is some support for this hypothesis. A comparison of reading and listening passage comprehension in 7- to 11-year-olds children with symptoms of ADHD, found that listening comprehension was more greatly impaired than reading comprehension (Cain & Bignell, 2014). However, recent longitudinal work found that grade 1 attention correlated only with grade 3 reading comprehension, not listening comprehension (Miller et al. 2014). Variability amongst studies may be, in part, due to the nature of the comprehension assessments (Johnston, Barnes, & Desrochers, 2008; Keenan & Meenan, 2014). For example, Miller et al. (2014) had a sentence cloze format, in which readers are required to fill in a word elided from a single sentence. Such tasks may make fewer demands on working memory and attention than passage comprehension, which requires the comprehender to construct a mental model. Indeed, a recent study by Kim (2016) that found an influence of both working memory and attention on concurrent listening comprehension (through other language skills) assessed listening comprehension with (narrative) passages. Here, we compared the relative contributions of both working memory and attention to parallel passage-based measures of reading and listening comprehension concurrently. Our goal was to understand better how these cognitive resources influence comprehension in the two modalities and if this differs by grade, which has educational implications for classroom practice and the development of grade level curricula.

### Direct and indirect relations to reading comprehension

Because word reading, working memory, and attention are each related to children’s reading comprehension, it is of interest to determine if the relation between working memory/attention and reading comprehension is direct or indirect. That is, is working memory/attention additional to the contribution made by word reading. Research to date broadly supports an indirect perspective. A study following children from kindergarten to grade 2 suggests an indirect relation between attention and reading comprehension, through the influence that attention has on the acquisition of word reading (Dally, 2006). Similarly, Miller et al. (2014) found that the relation between grade 1 attention and grade 3 reading comprehension was indirect, through the influence of attention on the acquisition of word reading, which then predicted subsequent reading comprehension.

The pattern of association between attention and reading comprehension for older children is less clear. A study of 7- to 11-year-olds found that word reading partly mediated the relation between attention and reading comprehension (Cain & Bignell, 2014) and there is evidence for a direct relation between attention shifting and reading comprehension in grade 4 (Kieffer, Vukovic, & Berry, 2013). The age differences may arise because the relative influence of word reading and listening comprehension on reading comprehension changes between grades 1 through 3 (Language and Reading Research Consortium 2015). Working memory influences the acquisition of oral language and supports the higher-level language skills critical for constructing a mental model of written or spoken text (Cain et al. 2004; Gathercole & Baddeley, 1993). Therefore, we examined the evidence for both direct and indirect relations between cognitive skills and reading comprehension in consecutive grades (1 through 3) and also longitudinally between grades 1 and 3 to provide a clearer picture of the nature of their relation.

## The current study

We examined the contributions made by working memory and attention to reading outcomes, both concurrently and longitudinally, to specify how working memory and attention contribute to reading and listening comprehension in the early grades. Children in grades 1 through 3 completed assessments of working memory, reading and listening comprehension, and word and nonword reading. Our assessments of working memory tapped both storage and processing of verbal information, rather than just simple short-term span, because these measures of ‘complex’ working memory have been shown to be strongly aligned with performance on national and standardized assessments of reading comprehension in previous research (Cain et al. 2004; Gathercole, Pickering, Knight, & Stegmann, 2004). Teachers and parents rated children’s overt behavioral attention (hereafter behavioral attention, see Miller et al. 2014, for a similar methodology). Our analyses extend the literature in the three important ways: a) we determine the individual and combined contributions of working memory and attention to measures of both reading and listening comprehension; b) we determine if these contributions are consistent or different across grades; and c) we determine if they influence reading comprehension directly, or indirectly through word reading, both concurrently and longitudinally. Critically, we used parallel passage-based measures of reading and listening comprehension because we were interested in the contribution of cognitive resources to the construction of the mental model of a text’s meaning. In line with the previous research, we predicted that working memory and/or attention would be more important for listening than for reading comprehension and that the relation between these cognitive skills and reading comprehension would be mediated by both listening comprehension and word reading ability.

## Methods

### Participants

Participants were drawn from a 5-year longitudinal study of reading comprehension [Language and Reading Research Consortium (LARRC)], which involved 915 children in preschool through grade 3 in Year 1 at four university sites (Arizona State University, University of Kansas, Ohio State University, and University of Nebraska-Lincoln). At each site, children were recruited through flyers sent home after contact was made with the individual schools and teachers. All children completed a battery of higher- and lower-level language, memory, listening and reading comprehension measures; their teachers and caregivers also completed surveys measuring the child’s attention skills and the classroom and home environments. For full details regarding the methods of the entire longitudinal study (see Language and Reading Research Consortium et al. 2016). For the purposes of this study, we report concurrent data on our variables from Year 1 of the study for children in grades 1 (*n *= 125), 2 (*n *= 123), and 3 (*n *= 122), and also longitudinal data from the grade 1 children 2 years later (when they were in grade 3). See Table 1 for demographic information for children in each grade in Year 1.

**Table 1:** Demographic characteristics of the sampled children

Characteristic	Grade 1	Grade 2	Grade 3
N	125	123	122
Age (baseline 2010)	6.56 (0.34)	7.53 (0.35)	8.58 (0.38)
Family income (categorical)			
% ≤ 40K	19.1	28.0	14.8
% 41K–80K	27.9	25.4	32.2
% > 80K	53.0	46.6	53.0
% Female	57	48	54
% White/Caucasian	81	86	75
% Hispanic	10	11	7
% FRL	16	26	16
% IEP	7	6	6
% English home language	78	86	77
Mother’s highest level of education			
% High school or lower	11.1	12.0	9.6
% Some college, AA/AS	21.4	27.3	30.8
% Bachelor’s degree	38.4	38.5	32.4
% Master’s or higher	29.1	22.2	27.2

### Procedures

Children were tested over the course of multiple sessions within a 5-month time frame (January to May). Measures were blocked together to make each testing session a reasonable length of time (60 min or less). All measures were administered by trained research staff in a quiet room in the child’s school, local university site, community center or home.

### Measures

Measures relevant to the present study are assessments of word and nonword reading, listening comprehension, reading comprehension, working memory and attention. We also report performance on nonverbal cognition to describe our sample. All standardized measures were administered according to the procedures described in the manual. Descriptive statistics are reported in Table 2.

**Table 2:** Mean raw^a^ scores, standardized^b^ scores (and standard deviations) by grade for observed variables

	Grade 1	Grade 2	Grade 3
Reading and listening comprehension			
Reading comprehension measure^a,c^	10.17 (3.19)	20.53 (4.74)	18.95 (4.66)
Listening comprehension measure^a,c^	12.04 (2.56)	19.47 (4.48)	20.65 (5.34)
Word reading			
WRMT-R: NU Word Identification^a^	49.25 (12.84)	59.89 (9.25)	68.52 (9.95)
WRMT-R: NU Word Identification^b^	119.23(11.73)	111.88 (9.94)	110.26 (10.31)
WRMT-R: NU Word Attack^a^	20.80 (8.49)	25.65 (8.17)	30.07 (7.81)
WRMT-R: NU Word Attack^b^	117.24 (9.43)	113.33 (13.80)	112.01 (13.82)
TOWRE Sight Word^a^	45.03 (14.55)	56.99 (10.08)	63.66 (10.87)
TOWRE Sight Word^b^	108.25 (15.18)	104.99 (12.28)	98.47 (13.29)
TOWRE Phonemic Decoding^a^	20.06 (10.53)	25.23 (9.38)	31.67 (11.68)
TOWRE Phonemic Decoding^b^	103.76 (14.47)	99.15 (12.75)	98.47 (14.84)
Working memory			
WJ III: Auditory Memory^a^	14.67 (5.21)	16.68 (5.25)	19.24 (5.61)
WJ III: Auditory Memory^b^	113.21 (14.40)	110.30 (14.65)	109.59 (16.01)
WJ III: Numbers Reversed^a^	8.80 (2.63)	9.69 (2.50)	11.20 (2.60)
WJ III: Numbers Reversed^b^	103.02 (14.49)	100.22 (13.63)	101.59 (13.14)
Memory Updating^c^	8.57 (3.73)	9.65 (4.19)	12.28 (4.58)
Attention			
SWAN: Attention teacher rating^c^	3.75 (1.35)	3.51 (1.34)	3.67 (1.32)
SWAN: Attention parent rating^c^	3.79 (0.92)	3.59 (0.96)	3.66 (0.99)
Descriptive variables			
KBIT-2^a^	106.32 (15.61)	108.93 (15.20)	109.19 (14.40)

*WRMT*-*R*-*NU* Woodcock Reading Mastery Tests-Revised: Normative Update, *TOWRE* Test of Word Reading Efficiency-2nd Edition, *WJ III* Woodcock Johnson III Test of Cognitive Abilities, *SWAN* Strengths and Weakness of ADHD-Symptoms and Normal-behavior, *KBIT* Kaufman Brief Intelligence Test, Second Edition

^a^Raw score

^b^Standardized score

^c^Standardized score not available

#### Word and nonword reading

Two subtests from the Woodcock Reading Mastery Tests-Revised: Normative Update (WRMT-R:NU; Woodcock, 1998) assessed accuracy of reading words and nonwords: Word Identification and Word Attack subtests respectively. In the Word Identification subtest participants read aloud real words. The reported split-half reliability is 0.98 for grades 1 and 2, and 0.97 for grade 3. Reliability for our sample (Cronbach’s α) was high = 0.96, 0.93, and 0.93, for grades 1 through 3. In the Word Attack subtest, participants read aloud pronounceable non-words. The reported split-half reliability is 0.94 for grades 1 and 2 and 0.91 for grade 3. Reliability for our sample (Cronbach’s α) was high = 0.92, 0.91, and 0.92, for grades 1 through 3.

Two subtests of the Test of Word Reading Efficiency-Second Edition (TOWRE-2; Torgesen, Wagner, & Rashotte, 2012) were administered to measure word reading fluency. The sight word efficiency (SWE) subtest measured how many printed English words, ranging from high to low frequency of occurrence, students could accurately pronounce in 45 s. The phonemic decoding efficiency (PDE) subtest assessed how many pronounceable non-words, varying in complexity, students could accurately pronounce in 45 s. The reported test–retest reliability for the SWE subtest is 0.93 and 0.91 for the PDE subtest.

#### Reading comprehension

Reading comprehension was assessed using the reading comprehension measure (RCM). This comprised six narrative and five expository passages and questions. Of these eleven passages, five were taken from the Qualitative Reading Inventory (QRI-5; Leslie & Caldwell, 2011), with some modifications, and six were created specifically for this project. All passages adhered to appropriate length and lexile level for each grade (according to the QRI manual). Participants read the passages (one expository and two narrative passages for grade 1; two of each type for grades 2 and 3) and then answered between 4 and 8 open-ended implicit and explicit questions, which together tapped the meaning-based representation of the text. Responses were audio-recorded and post-scored (0 or 1 point). The maximum total score differed by grade: 16, 30, and 28 points for grades 1, 2, and 3 respectively. Interrater reliability of scoring was good (0.93). Reliability for our sample (Cronbach’s α) was good = 0.76, 0.77, and 0.80, for grades 1 through 3.

#### Listening comprehension

Listening comprehension was assessed using the listening comprehension measure (LCM). This comprised six narrative and five expository passages and questions. Of these eleven passages, five were taken from the Qualitative Reading Inventory (QRI-5; Leslie & Caldwell, 2011), with some modifications, and six were created specifically for this project. All passages adhered to appropriate length and lexile level for each grade (according to the QRI manual). Children were presented with the same number of narrative and expository passages per grade as for the RCM. Responses were audio-recorded and post-scored (0 or 1 point). The maximum total score differed by grade: 16, 29, and 30 points for grades 1, 2, and 3 respectively. Approximately 10% of the sample from each grade was scored by a second examiner; interrater reliability was good (0.91). Reliability for our sample (Cronbach’s α) was adequate to good = 0.65, 0.75, and 0.83, for grades 1 through 3.

#### Working memory

Children completed three assessments of working memory. Two subtests from the Woodcock Johnson III NU Test of Cognitive Abilities (WJ III; Woodcock, McGrew, & Mather, 2001) were administered in which both storage and processing are required to perform the task successfully. In the Auditory Memory subtest, participants listened to the labels for a series of both digits and objects and were asked to reorder the series; first saying the objects in the order of presentation and then the digits in order of presentation. The reported test–retest reliability for ages 7, 8, and 9 were 0.84, 0.86, and 0.84, respectively. Reliability for our sample (Cronbach’s α) was good = 0.80, 0.82, and 0.84, for grades 1 through 3. In the Numbers Reversed subtest participants listened to an increasingly longer series of numbers and were asked to repeat the list backward. The reported test–retest reliability for ages 7, 8, and 9 were 0.90, 0.90, and 0.89, respectively. Reliability for our sample (Cronbach’s α) was adequate = 0.72, 0.69, and 0.70, for grades 1 through 3.

The Memory Updating task was based on Belacchi et al. (2010). This task measures the ability to modify the contents of working memory. Participants listened to a list of words and were asked to identify a specified number of the smallest items in the list (between 1 and 5 depending on list length). For example, “I want you to tell me the names of the two smallest things: fork, window, pig, shoe”. The number of words in the list increased from two to 12), as did the required number of items to recall. There were a total of five levels, with two lists of words within each level. If all attempts within a level were incorrect, the assessment was discontinued. The score was the total number of words correctly recalled. Reliability for our sample (Cronbach’s α) was good = 0.80, 0.79, and 0.82, for grades 1 through 3.

#### Behavioral attention

Classroom teachers and parents completed the Strengths and Weakness of ADHD-symptoms and normal-behavior scale (SWAN; Swanson et al. 2006), which comprised 18 statements regarding attention and also hyperactivity/impulsivity. There are 9 statements for each. The task is to respond about that child compared to others on seven-point scale (far below, below, slightly below, average, slightly above, above, far above) scored from 0 to 6, where 3 is equivalent to ‘average’. The mean score for each scale was computed: Inattention score and Hyperactivity/Impulsivity score. We include only the inattention scores in our analyses below (see Miller et al. 2014 and Kim, 2016, who used the same scale to measure attention). Examples of the (in)attention items involve assessment of overt inattentive behaviors, such as: *Often has difficulty sustaining attention in tasks or play activities; Often does not seem to listen when spoken to directly*. Reliability for our sample (Cronbach’s α) was high (> 0.91) for both scores.

#### Nonverbal cognition

Nonverbal cognition was assessed in the first year of the study only using the matrices subtest of the Kaufman Brief Intelligence Test, Second Edition (Kaufman & Kaufman, 2004) and is reported here to describe the general cognitive skills of our sample. This subtest measures problem solving abilities by determining how individuals perceive relationships and complete visual analogies. Internal reliabilities (reported in the manual) range from 0.78 to 0.93 (*M *= 0.88), depending on age. Reliability for our sample (Cronbach’s α) was high = 0.88, 0.87, and 0.85, for grades 1 through 3.

### Missing data

Missing data ranged from 4 to 10% for the outcome measures (i.e., LCM and RCM), and 0 to 6% for predictor variables. Instead of using listwise deletion, which has been shown to produce biased results and low power (Graham, 2012), we used full information maximum likelihood (FIML) to treat missing data in each step of the analyses (Arbuckle, 1996). FIML is a likelihood-based missing data treatment method that aims to directly estimate the values of model parameters using all information available. As an extension of the maximum likelihood (ML) method, the effectiveness of FIML is based on the adequacy of the hypothesized data model and the hypothesized missingness model. Therefore, in the context of multilevel modeling, when the hypothesized model is correctly specified, and the missing-at-random assumption (MAR, i.e., the distribution of missingness depends only on observed data) is plausible, the estimates derived from FIML should be unbiased (Little, Jorgensen, Lang, & Moore, 2014). While there is no conclusive way to prove that the data are MAR instead of not missing at random (NMAR), it is reasonable to make the assumption of MAR when there is a lack of ground to believe otherwise (Schafer & Graham, 2002). Moreover, in most applied research, departures from MAR are not so serious as to invalidate MAR-based techniques, such as FIML.

## Results

### Descriptive statistics of the sample

Table 2 reports the descriptive statistics of our measures. As is clear, general cognitive ability (nonverbal cognition) was slightly above average, but within the normal range for each grade. Note that when this score was included in the analyses to address our research questions (reported under the headings below) the pattern of prediction did not change. Standardized scores for the word reading and memory tasks (where available) show performance in line with average ability and is similar across grades. In general, performance was slightly better for listening than for reading comprehension. The same measures of word reading and working memory were administered to all three grades and, as expected, children from higher grades obtained higher scores than those from lower grades. The teachers’ and parents’ ratings of inattention were comparable across grades.

### Obtaining composite scores for predictors: word reading, working memory, and attention

Since we used multiple tests to measure word reading and working memory, we first conducted multi-group confirmatory factor analyses (CFA) to validate the measurement model, and to establish measurement invariance across grades. The final measurement models showed good fit and evidence of group invariance for both word reading and working memory (see Appendix 1), indicating that these two constructs are comparable across grades. Thus, we extracted factor scores of word reading and working memory from the multiple-group CFA model for further analyses. For the construct of attention, we computed the average score of teacher rating and parent rating, which are moderately correlated (G1: *r* = 0.575; G2: *r* = 0.616; G3: *r* = 0.509), in line with other studies (Swanson, 2011). The composite score was constructed such that larger values indicate higher levels of attention.

### What are the unique contributions of memory and attention to reading and listening comprehension and do they differ by grade?

Multilevel multivariate regression analyses were conducted using Mplus 7.11 (Muthén & Muthén, 1998–2012) for each grade separately. Multilevel modeling was used to account for the nested nature of the data (i.e., children were clustered by classrooms). The outcome variables were the standardized scores (z-scores) of listening comprehension and reading comprehension, which were allowed to correlate with each other in the multivariate model. Predictors were entered into the model in two steps. First, demographic characteristics including child’s age in months, gender (1 = girl), dummy-coded mother’s level of education (1 = bachelor’s degree or higher), and family annual income levels (Low income: 1 = family income ≤ $40,000; Middle income: 1 = family income between $40,001 and $80,000) were entered as the control variables. The original scales for maternal education and family income contained more than ten categories and were non-equidistant in nature, so a decision was made to dichotomize the variable of education and trichotomize that of income. Then the variables of interest, working memory and attention, were entered, and the additional percentage of variance accounted for (ΔR^2^) was calculated as a measure of the unique contribution of the key predictors. Table 3 summarizes the results of the regression analyses.

**Table 3:** The contribution of working memory and attention to RCM and LCM: multilevel multivariate regression analyses

	Grade 1 (*n* = 125)				Grade 2 (*n* = 123)				Grade 3 (*n* = 122)			
	RCM		LCM		RCM		LCM		RCM		LCM	
	B^a^	*p*	B^a^	*p*	B^a^	*p*	B^a^	*p*	B^a^	*p*	B	*p*
Step 1												
Age in months	− 0.00	0.999	− 0.02	0.777	0.16	0.010	0.07	0.396	0.13	0.253	0.04	0.709
Gender (1 = girl)	0.08	0.334	0.04	0.692	0.07	0.472	0.03	0.789	− 0.07	0.591	− 0.18	0.118
Mother having BA	0.27	0.006	0.25	0.023	0.05	0.671	− 0.02	0.851	0.31	0.001	0.13	0.239
Low income	− 0.19	0.047	− 0.11	0.363	− 0.15	0.188	− 0.24	0.025	0.00	0.967	− 0.08	0.493
Middle income	0.04	0.705	0.05	0.679	− 0.17	0.080	− 0.17	0.202	− 0.04	0.691	− 0.08	0.451
Step 2												
Working memory	0.24	**0.010**	0.34	< **0.001**	0.27	**0.016**	0.32	**0.003**	0.37	< **0.001**	0.47	< **0.001**
Attention	0.21	**0.023**	0.24	**0.006**	0.25	**0.012**	0.27	**0.001**	0.13	0.360	0.20	**0.020**
ICC (empty model)	0.241		0.005		0.029		0.033		0.169		0.093	
Residual variance^b^												
Step 1 (σ^2^, τ)	(0.808, 0.028)		(0.901, 0.022)		(0.904, 0.013)		(0.905, 0.016)		(0.770, 0.107)		(0.866, 0.058)	
Step 2 (σ^2^, τ)	(0.696, 0.031)		(0.706, 0.014)		(0.787, 0.007)		(0.746, 0.011)		(0.630, 0.134)		(0.654, 0.018)	
Child-level *R*^2c^												
Step 1	0.118		0.076		0.084		0.095		0.117		0.062	
Step 2	0.195**		0.261**		0.153**		0.208**		0.245***		0.316***	

Significant *p* values for the step 2 predictors (memory and attention) are shown in bold

*RCM* reading comprehension measure, *LCM* listening comprehension measure

^a^B = standardized coefficients

^b^σ^2^ = within-cluster (child-level) variance component; τ = between-cluster (classroom-level) variance component

^c^*p < 0.05; **p < 0.01; ***p < 0.001

For the first grade sample, the intra-class correlation (ICC) was 0.241 for reading comprehension, and 0.005 for listening comprehension, indicating that the majority of the variance in reading comprehension and listening comprehension lies among individuals. In other words, only 24.1% of variation in reading comprehension and 0.5% of variation in listening comprehension are attributable to classroom-level differences. The demographic characteristics accounted for 11.8% of the child-level variance in reading comprehension and 7.6% of the child-level variance in listening comprehension. After these controls, memory and attention together accounted for additional significant variance: 10.9% of variance in reading comprehension (*p *< 0.001) and 20.3% of variance in listening comprehension (*p *< 0.001).^1^ As expected, higher levels of working memory and behavioral attention were related with higher scores in reading comprehension and listening comprehension. Descriptively, working memory had stronger predictive power for listening comprehension (B = 0.34, *p *< 0.001, unique variance accounted for by listening comprehension = 8.8%) than reading comprehension (*B* = 0.24, *p* = 0.010, 3.8%). On the other hand, attention contributed similarly to reading comprehension (B = 0.21, *p* = 0.023, 2.8%) and to listening comprehension (B = 0.24, *p *= 0.006, 3.3%).

The results for second graders were broadly similar. We found that 97.1% of variance in reading comprehension (ICC = 0.029) and 96.7% of variance in listening comprehension (ICC = 0.033) lies among individuals. The demographic characteristics accounted for 8.4% of the child-level variance in reading comprehension and 9.5% of the child-level variance in listening comprehension. When considered next, working memory and behavioral attention together accounted for additional significant variance: 12.3% in reading comprehension (*p *< 0.001) and 16.4% in LCM (*p* < 0.001). Again, working memory was more predictive of listening comprehension (B = 0.32, *p* = 0.003, 7.7%) than of reading comprehension (B = 0.27, *p* = 0.016, 5.5%). Similar to what was observed among first graders, behavioral attention comparably contributed to reading comprehension (B = 0.25, *p *= 0.012, 4.2%) and to listening comprehension (B = 0.27, *p* = 0.001, 5.1%).

For third graders, 83.1% of variance in reading comprehension (ICC = 0.169) and 90.7% of variance in LCM (ICC = 0.093) was at the child level. Second, the demographic characteristics accounted for 11.7 and 6.2% of variation in reading comprehension and listening comprehension respectively at the child level. Third, working memory and behavioral attention together predicted additional and significant variance: 11.3% (*p *< 0.001) for reading comprehension and 25.2% (*p *< 0.001) for listening comprehension. When looking at working memory and behavioral attention separately, the results indicated a stronger contribution of working memory than attention to both reading and listening comprehension. Working memory was more predictive of listening comprehension (B = 0.47, *p *< 0.001, 16.5%) than of reading comprehension (B = 0.37, *p *< 0.001, 9.3%), and the same was true for behavioral attention (for reading comprehension, B = 0.13, *p* = 0.360, 0.0%; for listening comprehension, B = 0.20, *p* = 0.020, 2.6%). Different to the other samples, the unique contribution of behavioral attention to reading comprehension was not significant for third graders.

In sum, working memory and attention together accounted for between 11 and 12% of unique variance in reading comprehension, and 16 and 25% of unique variance in listening comprehension. For each grade, working memory was a significant predictor of reading comprehension and listening comprehension. Whilst working memory predicted somewhat greater variance in listening comprehension than reading comprehension, the magnitude of coefficients was not statistically different for the two measures, as demonstrated by the Wald test of constraints (*p* > 0.05). Although the test of model coefficients did not reveal any significant differences across grades, in terms of the contribution to total variance, working memory appeared to be most influential in third grade, where it uniquely accounted for 9% in reading comprehension and 17% of variance in listening comprehension, as compared to approximately 4–6% in reading comprehension in earlier grades and 8–9% in listening comprehension.

The pattern of prediction for behavioral attention was less consistent across grades. Behavioral attention uniquely predicted listening comprehension scores in all three grades, but it predicted reading comprehension scores only in the first and second grade. For both reading comprehension and listening comprehension, the unique contribution of attention was relatively stable across grades (0–4% for reading comprehension, 3–5% for listening comprehension).

### Is the influence of memory and attention on reading comprehension direct or indirect?

**Fig. 1:**
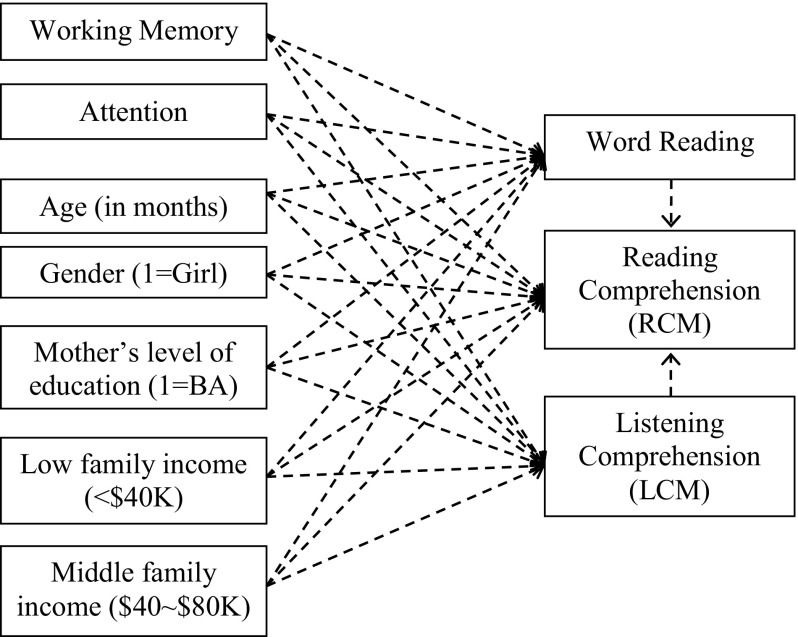
Path analysis: the mediating effect of word reading and listening comprehension

Multilevel path analyses were conducted for each grade to explore the relationships between working memory, behavioral attention, word reading, listening comprehension, and reading comprehension. Path analysis is a variant of structural equation modeling (SEM). It describes and tests a set of putative causal associations that are represented by a series of structural (i.e., regression) equations (Wright, 1934). In this case, with 370 participants nested within 121 classrooms, a multilevel path model was conducted in Mplus 7.11 to account for variation among classrooms and adjust for clustering. The outcome variable of interest was the standardized reading comprehension score, and other variables in the model were the demographic characteristics, working memory, attention, and word reading. It was hypothesized that, after controlling for the demographic variables, in addition to their direct contribution, memory and attention also contributed to reading comprehension scores indirectly through the mediation of word reading and listening comprehension, the two component skills involved in reading comprehension in accordance with the Simple View of Reading (Gough and Tunmer 1986). The diagram of this model is shown in Fig. 1 to allow for a clear conceptualization of the theory, and the results of the path analyses are summarized in Table 4.

**Table 4:** Multilevel path analysis: the direct and indirect effects of memory and attention on reading comprehension (RCM) through word reading and listening comprehension (LCM)

	Grade 1 (*n* = 125)		Grade 2 (*n* = 123)		Grade 3 (*n* = 122)	
	B^a^	*p*	B^a^	*p*	B^a^	*p*
Coefficients on mediators						
Memory → word reading	0.28	0.001	0.23	0.041	0.36	< 0.001
Attention → word reading	0.30	0.016	0.46	< 0.001	0.25	0.016
Memory → LCM	0.34	< 0.001	0.32	0.003	0.46	< 0.001
Attention → LCM	0.24	0.014	0.27	0.001	0.19	0.024
Direct effects on RCM						
Age in months	0.03	0.635	0.12	0.063	0.13	0.170
Gender (1 = girl)	0.06	0.378	0.04	0.608	0.00	0.972
Mother having BA	0.17	0.093	0.05	0.493	0.20	0.023
Low income (< $40K)	− 0.10	0.240	0.04	0.664	0.05	0.499
Middle income ($40K–$80K)	0.08	0.324	− 0.04	0.664	0.03	0.698
Word reading	0.21	0.021	0.16	0.124	0.38	0.001
LCM	0.40	< 0.001	0.57	< 0.001	0.38	< 0.001
Memory → RCM	0.05	0.652	0.05	0.589	0.05	0.580
Attention → RCM	0.05	0.529	0.01	0.928	− 0.03	0.797
Indirect effects on RCM						
Memory → word reading → RCM	0.06	0.040	0.04	0.196	0.14	0.009
Memory → LCM → RCM	0.14	0.015	0.18	0.009	0.17	0.005
Memory RCM (total indirect)	0.19	0.002	0.22	0.006	0.31	< 0.001
Attention → word reading → RCM	0.06	0.130	0.08	0.143	0.10	0.101
Attention → LCM → RCM	0.10	0.023	0.16	0.001	0.07	0.056
Attention RCM (total indirect)	0.16	0.001	0.23	0.003	0.17	0.028
Within R^2^ of RCM	34.3%		42.8%		44.1%	

^a^Standardized coefficients are indicated by B

Without adding any extraneous predictors, the intraclass correlation coefficients of the reading comprehension measure and the listening comprehension measure were the same as those obtained from the multilevel regression model (reported in Table 3). The ICCs of word reading were 1.2% for grade 1, 3.1% for grade 2, and 3.3% for grade 3, suggesting that the between-classroom variation in word reading is fairly small. After controlling for demographics, word reading, and listening comprehension, neither working memory nor attention had any significant direct effects on reading comprehension. With regard to the indirect effects, listening comprehension mediated the effects of working memory across all grade levels (grade 1: B = 0.14, *p *= 0.015; grade 2: B = 0.18, *p* = 0.009; grade 3: B = 0.17, *p* = 0.005), whilst word reading mediated its effects only in grades 1 and 3 (grade 1: B = 0.06, *p *= 0.040; grade 3: B = 0.14, *p* = 0.009). On the other hand, the effects of attention were mediated by listening comprehension for all grade levels (grade 1: B = 0.10, *p* = 0.023; grade 2: B = 0.16, *p* = 0.001; grade 3: B = 0.07, *p* = 0.056), albeit only marginal for grade 3, but were not mediated by word reading. Across the board, significant total indirect effects were observed for both working memory (grade 1: B = 0.19, *p* = 0.002; grade 2: B = 0.22, *p* = 0.006; grade 3: B = 0.31, *p *< 0.001) as well as attention (grade 1: B = 0.16, *p *= 0.001; grade 2: B = 0.23, *p* = 0.003; grade 2: B = 0.17, *p* = 0.028). Overall, 34–44% of the child-level variation in reading comprehension (grade 1: 34.3%; grade 2: 42.8%; grade 3: 44.1%) was accounted for by the path model.

The path models revealed that the contribution of working memory and attention was to a large extent mediated by word reading or listening comprehension. Specifically, working memory predicted word reading and listening comprehension, which in turn predicted the variance in reading comprehension. Attention predicted reading comprehension via the mediation of listening comprehension, but the indirect effect via word reading was not statistically significant.

### Are the longitudinal effects of memory and attention on reading comprehension direct or indirect?

**Fig. 2:**
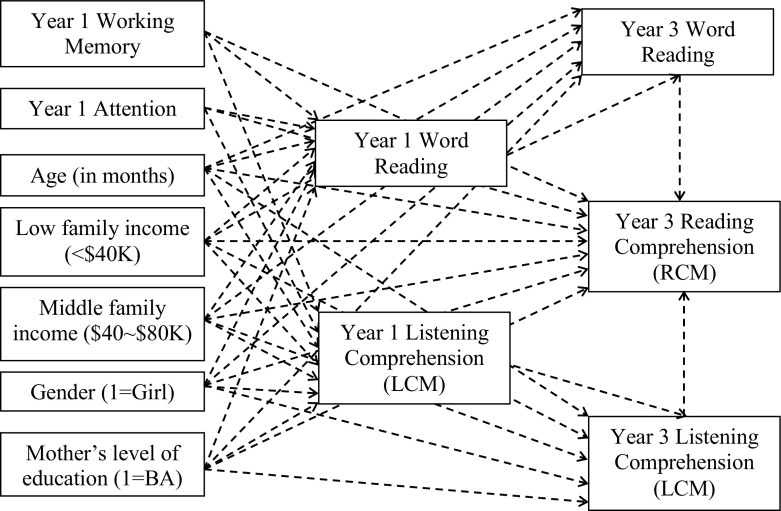
Longitudinal mediating effect of word reading and listening comprehension

We conducted multilevel path analyses to explore the longitudinal relationship between working memory and attention on reading comprehension. Specifically, we examined how performance in memory and attention tasks at grade 1 predicted reading comprehension outcomes 2 years later at grade 3 by testing whether (a) grade 1 memory and attention exerted a direct effect on grade 3 reading comprehension or (b) their influence was indirect through word reading and/or listening comprehension measured in grade 1 and then in grade 3. The first grade sample (*n* = 125) was used for this longitudinal analysis. The diagram of the hypothesized model is shown in Fig. 2, and the descriptive statistics for the Year 3 measures (RCM, LCM, and word reading factor score) are summarized in Table 5.

**Table 5:** Means and standard deviations of variables measured at Year 3 for grade 1 sample

	Grade 1 sample		
	N	Mean	SD
Year 3 listening and reading comprehension			
Reading comprehension measure	109	20.45	4.04
Listening comprehension measure	108	21.56	4.31
Year 3 word reading			
WRMT-R: NU Word Identification	110	69.05	8.88
WRMT-R: NU Word Attack	110	30.44	7.04
TOWRE Sight Word	110	65.55	9.53
TOWRE Phonemic Decoding	110	33.64	10.93

*WRMT*-*R*-*NU* Woodcock Reading Mastery Tests-Revised: Normative Update, *TOWRE* Test of Word Reading Efficiency

**Fig. 3:**
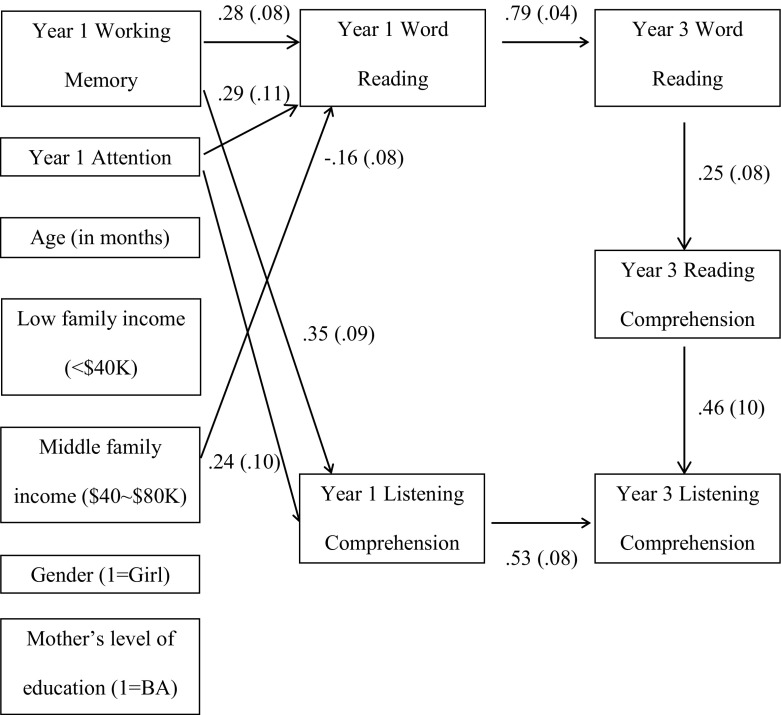
Longitudinal model displaying only significant paths (*p* < 0.05). *Note* Parameter estimates (standard error) are from the standardized solution

First, the intraclass correlation coefficient for the Year 3 reading comprehension measure was 0.147, indicating that 14.7% of variation in reading comprehension lies between classrooms in the third year, as compared to 24.1% of classroom-level variation when the same sample of children were in grade 1 (Table 3). Only a fraction of classroom-level variance was observed for the Year 3 listening comprehension measure (ICC = 0.016) and for the Year 3 word reading (ICC = 0.025). Based on the results of the path analyses (Table 6), there were no significant direct effects of grade 1 (Year 1) working memory or attention on grade 3 (Year 3) reading comprehension. The relationship between grade 1 memory/attention and grade 3 reading comprehension measure was mostly indirect, through the mediation of word reading and listening comprehension in a longitudinal fashion. Specifically, memory and attention were significant predictors of grade 1 word reading (memory: B = 0.28, *p *< 0.001; attention: B = 0.29, *p* = 0.006) and listening comprehension scores (memory: B = 0.35, *p *< 0.001; attention: B = 0.24, *p *= 0.015). These grade 1 scores respectively predicted word reading (B = 0.79, *p *< 0.001) and listening comprehension (B = 0.53, *p *< 0.001) at grade 3, which in turn predicted Grade 3 reading comprehension levels (word reading: B = 0.25, *p* = 0.003; listening comprehension: B = 0.46, *p *< 0.001). The total indirect effects were highly significant for both working memory (B = 0.14, *p *< 0.001) and attention (B = 0.12, *p *= 0.003). Overall, this path model accounted for 39.5% of child-level variation in Year 3 reading comprehension (Fig. 3).

**Table 6:** The direct and indirect effects of first grade memory and attention on third grade reading comprehension (RCM) through word reading and listening comprehension (LCM)

	Grade 1 sample (*n* = 125)	
	B^a^	*p*
Coefficients on mediators		
Y1 memory → Y1 word reading	0.28	0.001
Y1 attention → Y1 word reading	0.29	0.006
Y1 memory → Y1 LCM	0.35	< 0.001
Y1 attention → Y1 LCM	0.24	0.015
Y1 word reading → Y3 word reading	0.79	< 0.001
Y1 LCM → Y3 LCM	0.53	< 0.001
Direct effects on Y3 RCM		
Y3 word reading	0.25	0.003
Y3 LCM	0.46	< 0.001
Y1 memory → Y3 RCM	0.10	0.360
Y1 attention → Y3 RCM	0.12	0.268
Indirect effects on RCM		
Memory → Y1 LCM → Y3 LCM → Y3 RCM	0.09	0.010
Memory → Y1 WR → Y3 WR → Y3 RCM	0.06	0.031
Y1 memory Y3 RCM (total indirect)	0.14	< 0.001
Y1 attention → Y1 LCM → Y3 LCM → Y3 RCM	0.06	0.025
Y1 attention → Y1 WR → Y3 WR → Y3 RCM	0.06	0.024
Y1 attention Y3 RCM (total indirect)	0.12	0.003
ICC		
Y3 RCM	0.147	
Y3 LCM	0.016	
Y3 word reading	0.025	

*Y1* Year 1, *Y3* Year 3, *LCM* listening comprehension, *WR* word reading

^a^Standardized coefficients are indicated by B

## Discussion

We examined the extent to which working memory and teacher and parent ratings of behavioral attention predicted reading and listening comprehension in young readers in grades 1, 2, and 3 and also longitudinally between grades 1 and 3. Our working memory composite explained variance in both reading and listening comprehension and, as predicted, it explained a higher proportion of variance in listening comprehension. Developmental differences were also apparent: working memory was more important for both reading and listening comprehension in third grade than in the earlier grades; its effect on reading comprehension was mediated by listening comprehension in all grades, and by word reading in grades 1 and 3. Longitudinally, grade 1 working memory predicted grade 3 reading comprehension indirectly, through word reading and listening comprehension. The contribution of behavioral attention showed a broadly similar, but weaker, pattern: attention was more predictive of listening than reading comprehension in all grades; the effects of attention on reading comprehension were mediated by listening comprehension in all grades and by word reading in grades 1 and 2; longitudinally, the influence of attention on reading comprehension was indirect through word reading in grade 3, and listening comprehension in all grades. We first evaluate these findings in relation to previous research and then discuss their implications for both assessment and classroom practice.

Our findings demonstrate that the role of working memory in the prediction of comprehension is influenced by both modality and grade, although we must interpret these differences cautiously because differences between models were not statistically significant. We propose that the stronger role for listening than for reading comprehension is due to the different temporal demands of the two modalities as outlined earlier. Listeners with superior working memory skills will be better able to store and process accurately the meaning of each new piece of information and integrate its meaning into their mental model as the text unfolds. Readers, on the other hand, will be able to set the pace of delivery and re-read. As a result, their performance will be less influenced by individual differences in working memory, as demonstrated here.

Critically, our findings for indirect relations between working memory, behavioral attention and reading comprehension through their influence on word reading and listening comprehension align with other research (Dally, 2006; Miller et al. 2014). This finding is in line with other research demonstrating the critical importance of memory skills to the acquisition of word reading and oral language (Cain et al. 2004; Gathercole & Baddeley, 1993) and supports the view that cognitive skills are fundamental to our language and literacy development. To minimize poor reading comprehension outcomes, we must understand better the role of memory and attention in the acquisition of word reading and listening comprehension and support children with weaknesses in memory and attention in the early years classroom (Gathercole et al. 2016).

We propose that the greater influence of working memory with increasing grade is due to three factors. First, as texts become longer, with more complex syntactic structures in the later grades, language skills and the cognitive resources that support them will become increasingly influential (Adlof, Perfetti, & Catts, 2011). Thus, for both listening and reading comprehension, the influence of working memory may change over time. Second, both meta-analyses and empirical studies show that the relative influence of word reading on reading comprehension decreases and the relative influence of listening comprehension increases, during the first few years of reading instruction (Garcia & Cain, 2014; Language and Reading Research Consortium 2015) As a result, language comprehension skills, which are supported by working memory, will become more influential with increasing grade in determining reading comprehension outcomes. Third, performance on working memory updating tasks, which are related to the ongoing construction and refinement of the mental model in real time, continue to develop until at least adolescence (Linares, Bajo, & Pelegrina, 2016). Of note, we used memory materials that minimized the semantic load of the task; working memory tasks with a sentence comprehension component typically have a stronger relationship with reading and listening comprehension than other memory tasks (Nouwens, Groen, & Verhoeven, 2016; Seigneuric et al. 2000; Seigneuric & Ehrlich, 2005). Thus, our findings suggest that the link between working memory and text comprehension reported here is not solely due to variance shared with processing text for meaning.

The findings for behavioral attention are less clear and our explanation more speculative. Similar to the findings for working memory, behavioral attention predicted listening comprehension in all grades and was more important for listening than for reading comprehension. The effect of behavioral attention on reading comprehension was indirect for all grades. In contrast to working memory, behavioral attention predicted a smaller and nonsignificant proportion of unique variance in both reading and listening comprehension in grade 3 relative to grade 1, thus its effect was reduced with increasing age. One explanation for the weaker pattern of prediction by behavioral attention and a limitation of our study may be to do with the way that attention was assessed. Whilst we had multiple direct measures of memory, we had only parent and teacher ratings of behavioral attention. Thus, our memory composite might have better represented the multicomponential nature of the construct. In relation to this point, we note that memory updating had a lower loading than the other two (more similar) measures. There is good evidence that attention is not a unitary construct (Kieffer et al. 2013; Steele, Karmiloff-Smith, Cornish, & Scerif, 2012). Future research should include multiple direct measures of attention.

Another limitation of our study was that our memory measures were sensitive to developmental improvements, whilst our behavioral attention measure was a rating scale, designed to capture individual differences with an age group, not between grades. Thus, there was little variation in mean scores between grades. We recommend that future research uses direct measures designed to capture increasing attentional capacity both within and across development. Direct measures of attention would also ensure that that any relation between these and comprehension was not overestimated because teachers’ or parents’ ratings of behavioral attention might be influenced by their knowledge of a child’s comprehension skill. Furthermore, when teachers are asked to describe the characteristics of children with poor working memory, they often refer to poor attention, rather than poor working memory (Holmes et al. 2014). In addition, future work should explore the extent to which working memory and attention predict shared, as well as unique, variance in these skills given the moderate to strong relations that exist between performance on some working memory and attention tasks (Swanson, 2011). However, as noted by others, it may be hard to separate out the influences of memory and attention on language processing, because of their interdependence (Archibald, Levee, & Olino, 2015).

A strength of our study was the use of parallel measures of reading and listening comprehension: both measures involved passage-level comprehension, assessed by questions that tapped by literal and inferential information. For that reason, the differences between grades in the relative prediction by memory and attention that we find here cannot be explained by differences in the format of the assessments. However, we note that the listening comprehension measure in grade 1 was just adequate (0.65) and requires further refinement. On a theoretical note, future research should consider the prediction of literal vs inferential questions separately, to determine how they are differently predicted by memory and/or attention as suggested by previous research (Eason, Goldberg, Young, Geist, & Cutting, 2012). A methodological limitation is that we did not counterbalance our passages across reading and listening modalities. Future research seeking to replicate this finding should counterbalance materials to ensure that modality differences are not the result of systematic differences in materials.

We note that the ICCs for the reading comprehension measure differed (high in grades 1 and 3, but low in grade 2). We speculate that this difference arose because of the content of these passages: animal-based passages had a higher percentage of variance attributable to classroom level differences. One possibility is that certain classrooms (and books at home) may cover more animal-related contents than others. Indeed, of note the ICCs related to word reading were small, indicating perhaps a more uniform approach (or outcome) of instruction. Future research could usefully consider classroom topic content, given this finding, and the role of general knowledge in reading comprehension (Elleman & Compton, 2017).

Our use of passages to assess comprehension gave a fair test and allowed attention to have a role. Indeed, the use of passages is an additional strength of our study; our findings speak to the prediction of comprehension beyond the single word and sentence level, focusing on the mental model of the text. However, the use of a single measure of each is a limitation in the extent to which we can generalize our findings. The educational materials used in literacy classes and the assessment of written and spoken comprehension differ widely. In addition to the consideration of the content taught in classrooms noted above, further research could usefully explore the contributions of memory and attention to reading and listening comprehension measures that use different formats, to inform diagnosis and assessment (see also Johnston et al. 2008, for a discussion of this point), as well as comparing different text genres (narrative vs expository). Analyses by question type and text genre were outside the scope of this investigation.

We finish with the educational implications of our research. As noted, listening contexts may place additional cognitive load on comprehenders because the pace of delivery cannot be set to suit the individual, and material cannot be reviewed. Here, we found that cognitive resources were broadly more predictive of listening than of reading comprehension, at least in these early grades when word reading skills are developing. This suggests that delivery of classroom materials orally will not always be beneficial to the young beginner reader or one who struggles with word decoding. Second, the increasing influence of working memory may in part be due to changes in texts that align with increasing grade, such as length and more complex sentence structures as noted above. If so, then students in later grades with weak working memory skills may require additional support to access the curriculum. Third, the relation between working memory and comprehension confirms that early measurement of this cognitive resource is important as a marker of potential later reading comprehension and broader educational difficulties (Gathercole et al. 2003).

In sum, we found that the cognitive resources of working memory and attention are influential in the prediction of both reading and listening comprehension in the early grades and that their influence may be greater for listening than for reading comprehension. Further, we demonstrated that the influence of working memory and attention on reading comprehension is largely indirect through their influence on listening comprehension and word reading, both concurrently and longitudinally. Models of reading comprehension need to consider the role of these skills in the development of the component measures of the simple view. Future research could usefully employ a range of direct measures of attention to specify how and when it influences language comprehension to identify optimal classroom practice for beginner readers and those with literacy difficulties.

## Appendix 1

This paper was prepared by a Task Force of the Language and Reading Research Consortium (LARRC) consisting of Kate Cain (Convener), Kelly Farquharson, Hui Jiang, and Jill Pentimonti. LARRC project sites and investigators are as follows:

- Ohio State University (Columbus, OH): Laura M. Justice (Site PI), Richard Lomax, Ann O’Connell, Jill Pentimonti^1^, Stephen A. Petrill^2^, Shayne B. Piasta.
- Arizona State University (Tempe, AZ): Shelley Gray (Site PI), Maria Adelaida Restrepo.
- Lancaster University (Lancaster, UK): Kate Cain (Site PI).
- University of Kansas (Lawrence, KS): Hugh Catts^3^ (Site PI), Mindy Bridges, Diane Nielsen.
- University of Nebraska-Lincoln (Lincoln, NE): Tiffany Hogan^4^ (Site PI), Jim Bovaird, J. Ron Nelson.^5^

1. Jill Pentimonti is now at American Institutes for Research.
2. Stephen A. Petrill was a LARRC co-investigator from 2010–2013.
3. Hugh Catts is now at Florida State University.
4. Tiffany Hogan is now at MGH Institute of Health Professions.
5. J. Ron Nelson was a LARRC co-investigator from 2010–2012.

## Appendix 2

**Table:** Measurement invariance of word reading: fit statistics

	χ^2^ (df)	*p*	Δ χ^2^	*p*	CFI	RMSEA	Modification	Accept?
1. Configural invariance	3.34 (3)	0.343			1.000	0.030	No	Yes. Fit acceptable
2. Metric invariance	12.00 (8)	0.151	8.43	0.134	0.996 (− 0.004)	0.064	No	Yes. Fit comparable to 1
3.1 Scalar invariance	83.38 (14)	< 0.001	versus 2: 76.34	< 0.001	0.929 (− 0.067)	0.200	Partial scalar invariance^a^	No. Fit worse than 2, modify^a^
3.2 Partial scalar invar^a^	19.04 (12)	0.088	versus 2: 6.97	0.137	0.993 (− 0.003)	0.069	No	Yes. Fit comparable to 2

^a^Free the intercepts for Word Attack and Phonemic Decoding for Grade 1

**Table:** Measurement invariance of working memory: fit statistics

	χ^2^ (df)	*p*	Δ χ^2^	*p*	CFI	RMSEA	Modification	Accept?
1. Configural invariance	0.01 (1)	0.943			1.000	0.000	No	Yes. Fit acceptable.
2. Metric invariance	2.06 (4)	0.724	2.152	0.541	1.000 (− 0.000)	0.000	No	Yes. Fit comparable to 1
3. Scalar invariance	5.78 (7)	0.566	3.837	0.280	1.000 (− 0.000)	0.000	No	Yes. Fit comparable to 2

**Table:** Measurement model of word reading (partial scalar invariance across grade levels)

	Unstandardized loading	Standardized loading			*p*
		G1	G2	G3	
WRMT-R: NU Word Identification	1.000	0.812	0.851	0.890	< 0.001
WRMT-R: NU Word Attack	0.690	0.840	0.689	0.841	< 0.001
TOWRE Sight Word	1.000	0.989	0.897	0.852	< 0.001
TOWRE Phonemic Decoding	1.026	0.767	0.827	0.843	< 0.001

**Table:** Measurement model of working memory (scalar invariance across grade levels)

	Unstandardized loading	Standardized loading			*p*
		G1	G2	G3	
WJ III: Auditory Memory	1.000	0.517	0.548	0.576	< 0.001
WJ III: Numbers Reversed	0.457	0.447	0.520	0.569	< 0.001
Memory Updating	0.714	0.511	0.477	0.498	< 0.001

## Appendix 3

**Table:** Pearson correlation coefficients between key constructs

		1	2	3	4	5	6	7
Grade 1	1. Reading Comprehension	−						
	2. Listening Comprehension	0.535	−					
	3. Word Reading (factor score)	0.366	0.225	−				
	4. Working Memory (factor score)	0.363	0.445	0.437	−			
	5. Attention (average)	0.344	0.354	0.401	0.503	−		
	6. Teacher-Rated Attention Score	0.317	0.324	0.376	0.453	0.933	−	
	7. Parent-Rated Attention Score	0.292	0.260	0.317	0.404	0.846	0.575	−
Grade 2	1. Reading Comprehension	−						
	2. Listening Comprehension	0.646	−					
	3. Word Reading (factor score)	0.365	0.338	−				
	4. Working Memory (factor score)	0.356	0.401	0.342	−			
	5. Attention (average)	0.316	0.337	0.464	0.299	−		
	6. Teacher-Rated Attention Score	0.418	0.388	0.461	0.364	0.935	−	
	7. Parent-Rated Attention Score	0.073	0.149	0.336	0.147	0.868	0.616	−
Grade 3	1. Reading Comprehension	−						
	2. Listening Comprehension	0.519	−					
	3. Word Reading (factor score)	0.509	0.342	−				
	4. Working Memory (factor score)	0.401	0.516	0.434	−			
	5. Attention (average)	0.179	0.254	0.339	0.309	−		
	6. Teacher-Rated Attention Score	0.206	0.246	0.335	0.313	0.909	−	
	7. Parent-Rated Attention Score	0.109	0.208	0.235	0.204	0.824	0.509	−

## Footnotes

1 Since R^2^ change cannot be directly calculated in multi-level models, we computed the % additional variance as the reduction in the total residual variance when new predictors were added.
